# Towards Secure Big Data Analysis via Fully Homomorphic Encryption Algorithms

**DOI:** 10.3390/e24040519

**Published:** 2022-04-06

**Authors:** Rafik Hamza, Alzubair Hassan, Awad Ali, Mohammed Bakri Bashir, Samar M. Alqhtani, Tawfeeg Mohmmed Tawfeeg, Adil Yousif

**Affiliations:** 1 Institute for International Strategy, Tokyo International University, Saitama 350-1197, Japan; 2 National Institute of Information and Communications Technology, Tokyo 184-8795, Japan; 3 Department of Computer Science, School of Computer Science and Informatics, University College Dublin, Belfield, D04 V1W8 Dublin, Ireland; alzubair.mohamedtahir@ucd.ie; 4 Lero-the Irish Software Research Centre, Tierney Building, University of Limerick, Sreelane, V94 NYD3 Limerick, Ireland; 5 Department of Computer Science, College of Science and Arts—Sharourah, Najran University, Sharourah 68341, Saudi Arabia; aaomar@nu.edu.sa; 6 Department of Math, Turubah University College, Taif University, Taif 26571, Saudi Arabia; safi@tu.edu.sa; 7 Department of Computer Science, Faculty of Computer Science and Information Technology, Shendi University, Shendi 41601, Sudan; 8 Department of Information Systems, College of Computer Science and Information Systems, Najran University, Najran 61441, Saudi Arabia; smalqhtani@nu.edu.sa; 9 Department of Computer Science, Faculty of Computer Science and Information Technology, University of Science and Technology, Khartoum 14411, Sudan; tawfeeg.mohammed@ust.edu.sd

**Keywords:** big data, encryption algorithms, homomorphic encryption, privacy preserving, machine learning

## Abstract

Privacy-preserving techniques allow private information to be used without compromising privacy. Most encryption algorithms, such as the Advanced Encryption Standard (AES) algorithm, cannot perform computational operations on encrypted data without first applying the decryption process. Homomorphic encryption algorithms provide innovative solutions to support computations on encrypted data while preserving the content of private information. However, these algorithms have some limitations, such as computational cost as well as the need for modifications for each case study. In this paper, we present a comprehensive overview of various homomorphic encryption tools for Big Data analysis and their applications. We also discuss a security framework for Big Data analysis while preserving privacy using homomorphic encryption algorithms. We highlight the fundamental features and tradeoffs that should be considered when choosing the right approach for Big Data applications in practice. We then present a comparison of popular current homomorphic encryption tools with respect to these identified characteristics. We examine the implementation results of various homomorphic encryption toolkits and compare their performances. Finally, we highlight some important issues and research opportunities. We aim to anticipate how homomorphic encryption technology will be useful for secure Big Data processing, especially to improve the utility and performance of privacy-preserving machine learning.

## 1. Introduction

A major challenge for large companies and enterprises today is ensuring data security and privacy when processing large amounts of data. Most IT companies collect, transport, store, and analyze large amounts of data and face significant data protection problems on a daily basis. These problems make the realization of the “Society 5.0” project a tricky matter. The issues of data privacy in transit and at rest have attracted much attention in recent decades [1]. There are now cryptographically based security mechanisms that securely and effectively protect data sets of any size in transit over the network and in storage in the data repository, such as homomorphic encryption and the use of blockchain technology [2,3]. However, the unresolved issue is how to effectively and securely protect the privacy of the collected data during its processing.

Private preserving set intersection computing is a fundamental function in many big data applications, e.g., medical and financial [4,5]. It is a high priority to preserve information security and privacy protection in the area of cross-big data analytics applications. In this case, datasets are mainly encrypted before uploading, transmission, and processing. In this case, a user encrypts his data by using his secret key and sends the encrypted ciphertext to the analytic service so that all the user data will be encrypted and stored on the cloud servers. Herein, without decrypting the user’s data, statistical analysis could not be computed without the need to exchange the user’s secret keys or information attached to their data. Due to the confidential nature of some private information assets, only limited authorized users, including the data owners, can access and use these information assets. Yet, these private information assets can contribute to increasing the accuracy of the results of any machine learning models and the precision of the analytics mechanisms [6]. In this regard, scientists suggest that collaboration between cryptography and big data analysis techniques will allow the safe and fair utilization of private information assets empowered by machine learning techniques.

Previous studies have identified standardization challenges and implementation issues as shortcomings of previous research implementing homomorphic encryption algorithms to preserve the privacy of sensitive big data analysis [7,8]. Previous research has concentrated principally on the improvement of innovative mechanisms, protocols, and proofs of concept toward using homomorphic encryption algorithms with big data. Big data in real life can be represented by heterogeneous datasets. This could create different challenges for complex real-world big data applications. In this regard, homomorphic encryption is not only used well in scientific computing, but is also utilized in privacy-protecting computing to complete related data sets [9].

**Figure 1:**
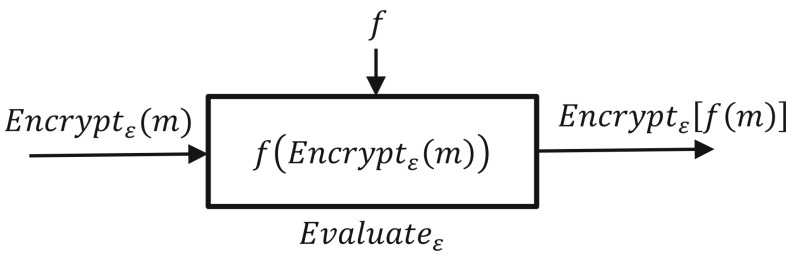
Homomorphic encryption concept [13].

**Figure 2:**
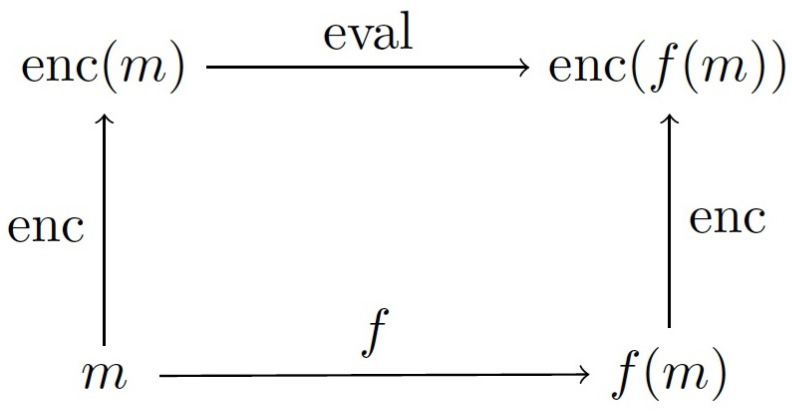
Fully homomorphic encryption structure.

Homomorphic encryption technology is a game-changing new technique that provides private cloud storage and computing solutions, and numerous applications have been detailed in the literature in recent years [10,11,12]. Homomorphic encryption algorithms are currently being widely deployed with various applications to secure users’ data and their privacy, including in the medical, industrial, and financial sectors [4,5,12]. Figure 1 and Figure 2 show the main concept of homomorphic encryption, which can perform data operations over an encrypted domain. Indeed, homomorphic encryption has been acknowledged as one of the most effective methods for protecting and processing data through remote servers, including the cloud. However, before homomorphic encryption can be widely used, it must be standardized, most likely by a number of standards groups and government agencies. There is a comprehensive understanding of security levels that is based on the distinct parameter sets as a fundamental element of standardization for the homomorphic encryption algorithms [7,8]. Although the academic community has conducted considerable study and benchmarking to lay the groundwork for this endeavor, it is difficult to obtain all of the facts in one place along with guidelines for big data applications and implementation.

In this paper, we present a detailed overview of all the major homomorphic encryption algorithms for big data analytics. We present a cutting-edge overview of homomorphic encryption’s major tools and applications in big data analytics using machine learning and statistical analysis. We discuss the capabilities and challenges of homomorphic encryption in an industrial context, with a focus on privacy-preserving machine learning computation. Herein, we intend to predict how homomorphic encryption technology will be useful for the secure processing of big data, particularly in increasing the utility and performance of privacy-preserving machine learning for big data analytics. Furthermore, we present the results of the implementation of different homomorphic encryption libraries and compare the execution time performances. Finally, we highlight some discussion and research opportunities with regard to the future of homomorphic encryption algorithms. This survey is designed to provide researchers and practitioners with a clear understanding and basis for comprehending, applying, and expanding relevant state-of-the-art homomorphic encryption algorithms for big data analytics.

The rest of this article is arranged as follows: In Section 2, characterizations of different homomorphic encryption algorithms, including partial, somewhat, and fully homomorphic encryption algorithms, are presented. In Section 3, we highlight some important points with regard to homomorphic encryption security. Then, in Section 4, we discuss cases of homomorphic encryption for large data, followed by Section 5, which examines the implementations of available homomorphic encryption toolkits along with relevant results and evaluation. Section 6 provides a discussion of the challenges of using homomorphic encryption algorithms. Finally, the article is concluded in Section 7.

## 2. Preliminaries

In this section, we present some basic mathematical and cryptographic definitions related to the homomorphic encryption algorithms, to which the interested reader should refer to the references [14,15].

More generally, a homomorphic encryption algorithm ϵ is a tuple of algorithms $\left(KeyGen_{\epsilon }\left(\lambda \right),Enc_{\epsilon }\left(pk,M\right),Dec_{\epsilon }\left(sk,\Psi \right),Eval_{\epsilon }\left(pk,f,\Psi \right)\right)$, and $f\in F_{\epsilon }$ belongs to the family of admissible functions where

- $KeyGen_{\epsilon }\left(\lambda \right)$ is the key generation method that takes the security parameter lambda as input and provides the couple (*pk*, *sk*), public key and private key, accordingly.
- Encepsilon(pk,M) is an encryption algorithm that uses the public key pk to encrypt the plaintexts $M=(m_{1},...,m_{n})$ from the ring of plaintexts *P* and produces a set of cyphertext Psi=(c1,...,cn)
- $Dec_{\epsilon }\left(sk,\Psi \right)$ is the decryption algorithm that collect cyphertexts Psi and returns a set of plaintexts *M* using the secret key sk.
- $Eval_{\epsilon }\left(pk,f,\Psi \right)$ gather the public key and execute the evaluation of the function *f* on Ψ. This algorithm returns a set of cyphertexts $\bar{\Psi }$.

Note it is possible to define an encryption algorithm as homomorphic over a mathematical process ⋄ which is defined on a ring of plaintexts *P* if: $Enc_{\epsilon }\left(pk,m_{1}⋄m_{2}\right)=Enc_{\epsilon }\left(pk,m_{1}\right)\circ Enc_{\epsilon }\left(pk,m_{2}\right)$

for ∘ established on a ring of ciphertexts *X*. Moreover, it is fundamental: $m_{1}⋄m_{2}=Dec_{\epsilon }\left(sk,c_{1},c_{2}\right)$

### 2.1. Partially Homomorphic Cryptosystems

**Figure 3:**
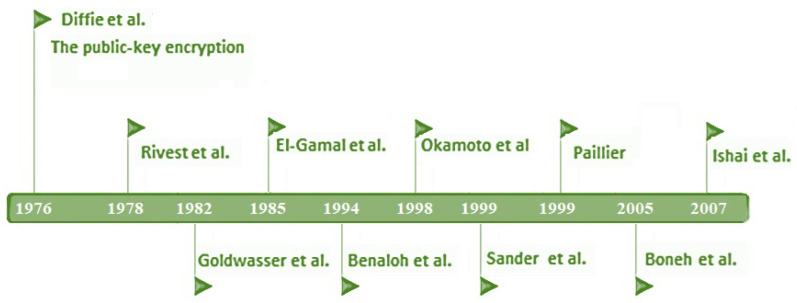
Timeline of homomorphic encryption algorithms leading up to Gentry’s first fully homomorphic encryption.

A cryptographic algorithm is defined as partially homomorphic if it can perform additive or multiplicative homomorphism but not both operations at the same time. Figure 3 shows the timeline of homomorphic encryption algorithms leading up to Gentry’s first fully homomorphic encryption. Partially homomorphic encryption algorithms are, in general, more efficient than fully and somewhat homomorphic encryption algorithms [16]. Herein, RSA, ElGamal algorithms [17,18] with multiplicative homomorphism, and Paillier additive homomorphism [19], respectively, are well-known examples of partial homomorphic algorithms. Many big data have been proposed in the last few years based on partial homomorphic algorithms such as in healthcare [20], intelligent transportation systems [21], and deep learning applications [2,22].

#### 2.1.1. Multiplicative Partial Homomorphic Encryption: Unpadded RSA

In the case of RSA, if the encryption algorithms have a public key with modulus nn and encryption exponent ee, then the encryption operates on a message mm which is given mathematically by $E\left(m\right)=m^{e}\;\;\mathrm{mod}\;\;nE\left(m\right)=m^{e}\;\;\mathrm{mod}\;\;n.$ The homomorphic property is then

$$\begin{array}{cc} E\left(m_{1}\right)\cdot E\left(m_{2}\right) & =m_{1}^{e}m_{2}^{e}\;\;\mathrm{mod}\;\;n\\ & ={\left(m_{1}m_{2}\right)}^{e}\;\;\mathrm{mod}\;\;n\\ & =E(m_{1}\cdot m_{2}) \end{array}$$

#### 2.1.2. Additive Partial Homomorphic Encryption: Goldwasser–Micali

If this algorithm uses the public key with a modulus nn and quadratic non-residue xx, thereafter, the encryption of bb is $E\left(b\right)=x^{b}r^{2}\;\;\mathrm{mod}\;\;n$, for some random r∈{0,…,n−1}. Accordingly, the homomorphic property is

$$\begin{array}{cc} E\left(b_{1}\right)\cdot E\left(b_{2}\right) & =x^{b_{1}}r_{1}^{2}x^{b_{2}}r_{2}^{2}\;\;\mathrm{mod}\;\;n\\ & =x^{b_{1}+b_{2}}{\left(r_{1}r_{2}\right)}^{2}\;\;\mathrm{mod}\;\;n\\ & =E(b_{1}⊕b_{2}). \end{array}$$

where ⊕ is defined as addition modulus two. Table 1 lists well-known partially homomorphic encryption algorithms’ operations and properties.

### 2.2. Fully Homomorphic Cryptosystems

**Figure 4:**
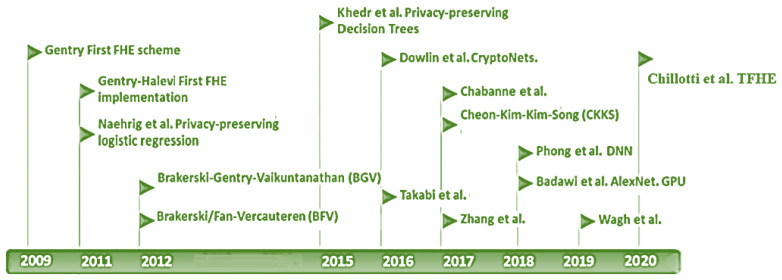
Fully Homomorphic Encryption Timeline.

A cryptographic algorithm is defined as fully homomorphic if it has both additive and multiplicative homomorphism operations [23]. In theory, fully homomorphic encryption is referred to as a class of encryption algorithms anticipated by Rivest in 1978 [18], and the first full homomorphic system was developed by Craig Gentry in 2009 [23]. Gentry scheme relies on a complicated mesh of ideal lattices to represent the pair of keys and the ciphertext. Later, many researchers and industrial companies worked on Gentry’s proposal, addressing the obstacles to having a more practical algorithm. Figure 4 shows the fully homomorphic encryption categories after the breakthrough of innovative research by Gentry. Until now, all efforts have been directed toward developing new efficient and effective fully homomorphic algorithms that address the challenges of lattice-based encryption algorithms [8,23,24,25].

Gentry proposed a bootstrapping approach to make the encryption fully homomorphic. The bootstrapping part could be used for bootstrapable ciphertexts noise-based with a short circuit measurement [23,26]. The maximum number of operations should be proportional and equivalent to the depth of the circuit.

Gentry’s bootstrapping phase is only permitted for decryption operations with a low depth. As a result, he utilized certain “tweaks” to lower the complexity of the decryption methods. This technique is known as “squshing”. In fact, Gentry’s technique implies selecting a collection of vectors whose sum matches the secret key’s multiplicative inverse. When the ciphertext is multiplied by the set’s elements, the polynomial degree of the circuit is lowered to a level that the algorithm can manage. Herein, the ciphertext should be “bootstrappable”. Moreover, the difficulty of retrieving the private key is defined based on the Sparse Subset Sum Problem (SSSP) [27], which illustrates the provable security of the algorithm.

Bootstrapping is fundamentally a “decryption” technique that produces a “clear” ciphertext from the noise-based encryption matching the original plaintext. If an algorithm can assess its own decryption circuit, it is said to be bootstrappable [23]. First, the ciphertext is converted into a bootstrapable ciphertext via a squashing algorithm. Then, using the bootstrapping technique, a “clear” ciphertext is obtained. The following is how bootstrapping works: First, it is expected that two distinct pairs of public and secret keys are produced. Herein, the client keeps the secret keys while the server shares the public keys. The secret key is then encrypted and sent to the server, which already possesses the encrypted plaintext (ciphertext). The noisy ciphertext is decrypted homomorphically since the abovementioned relative encryption technique will evaluate and assess its possessed decryption procedure homomorphically. The result is then encrypted with a second, different public key. An adversary cannot distinguish between the encryption of the secret key and the encryption of an arbitrary number, since the algorithm is considered semantically secure [26]. The final ciphertext is decrypted using the client’s second private key, which should be kept secret by the client. In summary, noisy ciphertext is homomorphically decrypted to eliminate the noise, then the distinct homomorphic encryption creates additional tiny noise in the ciphertext. The ciphertext is now just encrypted data. Further homomorphic computations on this “clear” ciphertext can be calculated until a threshold point is reached.

Fully homomorphic encryption algorithms can give a third party the means to fully execute arbitrary computations on encrypted plaintext as needed and without learning any part of the inputs or the computation results. While there are benefits to secure computation using fully homomorphic encryption algorithms, they have some overhead disadvantages. For example, Gentry’s bootstrapping approach significantly raises the computing cost and becomes a key disadvantage for the feasibility of fully homomorphic encryption.

### 2.3. Somewhat Homomorphic Cyptosystems

There are a number of cryptosystems that have been defined as somewhat homomorphic and can support specific operations in a restricted number of applications. Somewhat homomorphic schemes support simultaneous addition and multiplication where the highest number of permitted homomorphic operations are restricted [28,29].

Even though fully homomorphic encryption can be implemented and realized, most practical deployments apply tiered or somewhat homomorphic encryption algorithms [30]. This is because somewhat homomorphic encryptions have restrictions on the multiplicative depth of the circuits they can assess and dodge computationally exhaustive bootstrapping.

In the last few years, many fruitful somewhat homomorphic cryptosystems have been presented and discussed in the literature [31,32,33,34]. Gentry’s [23] publication of the first feasible fully homomorphic encryption algorithm marked a turning point for somewhat homomorphic cryptosystems. In addition to the Gentry algorithm, there are some important somewhat homomorphic cryptosystem versions that were also proposed [35,36]. Many researchers rely on somewhat homomorphic algorithms rather than other techniques, primarily because of problems with the performance of fully homomorphic encryption schemes. In the following section, we focus largely on the primary schemes used as a step toward the first practical scheme of homomorphic cryptosystems.

## 3. Homomorphic Encryption Security

A homomorphic encryption algorithm will provide a high level of data security to the applications that use it, and standardization will increase its prevalence and usefulness. A key component of this standardization process will be a consensus on the level of security parameters in different implementations and systems. According to many references, a homomorphic encryption algorithm has three security properties [37]:

1. No adversary can determine whether two different messages have been encrypted from any given ciphertext. Here, the encryption is randomized to guarantee that the same message cannot have the same encryption, and this guarantees semantic security.
2. Compactness: homomorphic encryption operates on ciphertext and does not extend the length of the ciphertext.
3. Efficient decryption and operation on the ciphertexts. The runtime of restoring the plaintext should be independent of the functions that were derived from the ciphertexts. As a result, the functions in the ciphertexts do not depend on the decryption algorithm.

Furthermore, the homomorphic encryption algorithms have different parameters. Overall, these settings and parameters demand four inputs:

- λ: a security level. For instance, 128-bit security (λ = 128) or 256-bit security. This is a desired security level of the encryption algorithm where increasing the λ will increase the level of security.
- A plaintext modulus P depends on the chosen homomorphic encryption algorithm. This standard currently identifies two categories of parametrized plaintext spaces: modular integers and extension fields/rings. The interested reader may refer to [37,38].
- A dimension K for the vectors that need to be encrypted.
- The auxiliary parameter B is used to control the complexity of the homomorphic scheme that can be used to perform the encryption operation. Lower parameters result in significant improvements that are ”smaller” or less expressive or require minimally complex programs/circuits. Lower parameters generally mean that the parameters of the entire system are smaller. As a result, ciphertexts are smaller and evaluation procedures are more efficient. On the other hand, higher parameters generally increase the size of the key, the size of the ciphertext, and the complexity of the evaluation procedures. Higher parameters are obviously necessary to evaluate more complex programs. In order to analyze more and more sophisticated programs, higher parameters are always required [23,37].

These parameters are utilized to create the pairing key for encryption and decryption (a public key and a secret key) and an evaluation key. Anyone can use the public key to encrypt a message, while only authorized users can use the secret key for the decryption process. The evaluation key should be sent to an authorized party to decrypt the data.

Recent research has shown that homomorphic encryption schemes are not secure against a chosen-ciphertext attack (CCA) and that the existing homomorphic encryption schemes achieve weaker indistinguishability under a chosen ciphertext attack (IND-CCA) security condition [39,40]. The structure of a fully homomorphic encryption scheme is not provably secure and contains implicit assumptions about the interplay between these underlying primitives. Accordingly, it is an acceptable hypothesis to assert that a homomorphic encryption scheme alone cannot provide secure outsourced computation. Due to the lack of CCA security, the system must guarantee that decryption is never performed with incorrect ciphertexts. Allowing the attacker to submit erroneous ciphertexts for decryption often leads to the disclosure of the secret key in modern homomorphic encryption methods. Therefore, with homomorphic encryption methods, it is common to settle for CPA security and rely on the system around the homomorphic encryption to provide the additional protection that may be required for any application.

Recently, Li and Micciancio [41] discovered that approximate-number homomorphic encryption systems are vulnerable to some known attackers. The commonly held notion of CPA security may even be inadequate against passive attackers. The key difference is that the attacker might learn something from the decryption results because the algorithm itself introduces some errors. If the attacker discovers the error, he can obtain information about the secret key. Li and Micciancio have developed a simple attack on the CKKS approach using approximate values that reveals the secret key after only a few decryption results. The attack shows that the usual definition of IND-CPA security (or indistinguishability against selected plaintext attacks) produced by CKKS does not effectively include security against passive attackers when applied to proximity encryption systems. The authors concluded that a separate, stronger definition is needed to evaluate the security of such systems.

This attack approach is only suitable for scenarios where the adversary only has access to a set of decryption results. For example, the HElib Library [42] suggests that to avoid an attack that has access to the decryption results of D ciphertexts, the bit parameter should generally be increased by a factor of D and the input precision parameter should be similarly increased. The HElib Library suggests that if the adversary has access to a significant amount of information about decryption requests, the application must increase the bit parameter from above. Further security assessments of the initial values are required, and each industry organization should pay attention to the security level of each homomorphic encryption library.

## 4. Use Cases of Homomorphic Encryption for Big Data Applications

**Figure 5:**
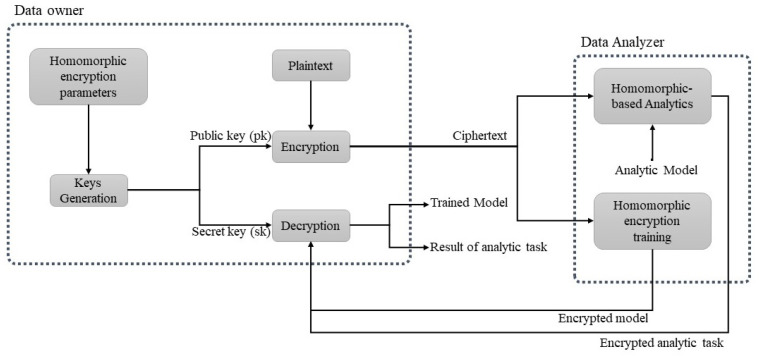
Private prediction and private training services.

To enrich this research, we present a practical scenario in which homomorphic encryption is used for privacy-preserving machine learning algorithms for Big Data analytics. Figure 5 shows the details of this scenario. For example, imagine an untrusted data analyzer that has a trained model and perhaps computational capabilities to perform the analysis tasks for various users’ data. Originally, the private prediction was introduced as a service when the data owner outsourced the analysis or prediction of private encrypted data psi to a third party.

The main goal of various homomorphic encryption algorithms is to achieve unconstrained and practical encryption deployment. We focus on a complex, fully homomorphic encryption implementation for real-world Big Data analytics. Unlike previous theoretical methods, which are either proof-of-concept methods or restricted deployments and implementations, Gentry et al. have constructed a fully homomorphic encryption for the first time to evaluate a circuit complicated enough for a practical real-world implementation. The focus is on deploying database server and client security using the homomorphic encryption structure as described in the literature [3,43,44,45]. The fully homomorphic encryption algorithm’s computing process is depicted in Figure 5.

The problem of privacy-preserving big data has been investigated for years [28,46,47]. Various privacy-preserving protocols have been proposed. Generally speaking, privacy-preserving big data protocols can be classified into two categories: randomization-based approaches and secure multiparty computation (SMC)-based approaches. Due to the research target of our paper, we will only discuss secure multiparty computation via homomorphic encryption. The interested reader may refer to [48,49]. The secure multiparty computation-based approach is often used to develop ways in which participants can collectively compute a function, using their information while maintaining privacy.

Two possibilities between the data owner and data analyzer are explored, as shown in Figure 5. In this context, the first phase considers private prediction as a service, so that a data owner can outsource the analysis of its encrypted data psi via a cloud or a third party. As an example, consider an untrusted data analyzer that has a trained model and perhaps the computational capacity to perform the prediction. The training service is considered as the second phase, where the owner provides the ciphertext psi to the cloud to deliver an encryption-ready model $w_{encr}$ and then $w_{encr}$ to perform analysis on new ciphertexts. The created model is ready for the analysis requests. In both cases, the encrypted results are returned to the data owner for decryption. In the first situation, the data owner knows only the result of the data prediction $P_{encr(psi)}$. Nothing is known about the model *w*, which may be a secret property for the untrusted data analyzer.

Gentry et al. [50] implemented an extension of the one proposed by Brakerski et al. [24] proposed BGV algorithm. The authors performed this study using a leveled FHE without bootstrapping from the BGV algorithm to homomorphically evaluate the AES circuit. Gentry et al. [50] used a leveled full homomorphic encoding without bootstrapping and a version of the algorithm presented by Brakerski et al. [24] to homomorphically score the AES circuit. The main advantage of using AES is speed. A symmetric key method consumes less processing resources and runs faster and more efficiently. In fact, the concept of homomorphic evaluation of AES was first introduced in Naehrig et al. [38] with the following situation explored. A client supplied the key AES by encrypting it with a full holomorphic encryption method, abbreviated FHE in this context. The client then transmits the data while encrypting it using only AES, and AESK (m). If the cloud decides to assess the data in a homomorphic manner, it could perform FHE (AESK (m)) and decrypt AES homomorphically (blindfold) to proceed with FHE (m). The cloud may then conduct any homomorphic execution on the FHE-encrypted data.

Smart and Vercauteren [51] suggested employing homomorphic evaluation of AES to perform SIMD (single-instruction multiple-data) computations. Later, some studies [14,51,52] enhanced the performances of the AES circuit’s homomorphic evaluation by using current theoretical enhancements and optimizations.

**Figure 6:**
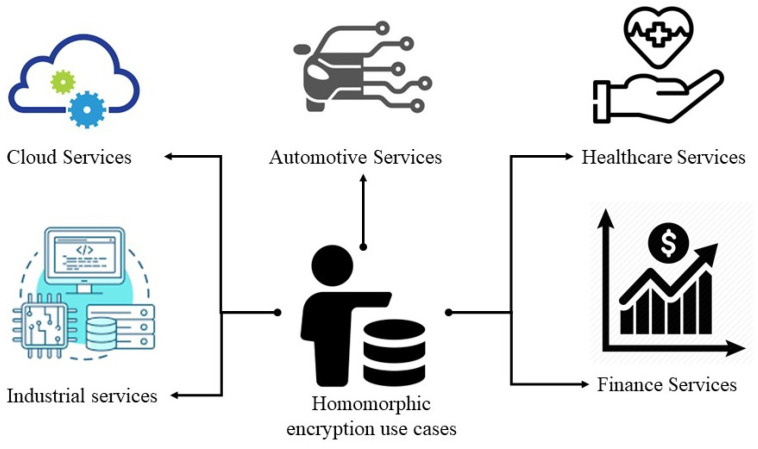
Use cases of homomorphic encryption for big data.

Multi-key homomorphic encryption can deal with the use cases of multiple parties [48,53], combining their separately encrypted data in one service, then processing the encrypted data, and finally, only the authorized parties that provided the data could see the outcome. The use cases can be developed further with the adoption of multi-key homomorphic encryption. Figure 6 shows some potential homomorphic encryption real-world use cases, especially with the adoption of a multi-key mechanism. We illustrate some examples of industrial viable use cases where homomorphic encryption can be deployed directly, such as customer financial and credit risk rating [54,55], supply chain [56], automotive systems [57], and healthcare applications [58,59].

The literature provides numerous examples of the use of homomorphic encryption algorithms in in-depth case studies. For example, Guo et al. [60] addressed the problem of recognition speed on the circuit board of a robot and security issues in the cloud. Morampudi et al. [61] presented a privacy-friendly iris authentication with fully homomorphic encryption. The authors proposed a new mechanism for iris authentication and enforced the security level using the fan-vercauteren scheme [62]. Lagesse et al. [63] developed and implemented a Raspberry-based framework for similar video detection and effectively worked with a fully homomorphic encryption algorithm in mobile device environments.

Introducing homomorphic libraries with Big Data applications can achieve pure data analyzers with a minimum of cryptographic experience while providing a smooth and familiar interface and API. Most privacy-friendly machine learning technologies use homomorphic encryption libraries to ensure pre-processing capability and security of private information. In this context, Cape Privacy Company has developed TF Encrypted [6], a TensorFlow-based framework. TensorBoard allows users to explore the static data flow graph, which helps identify and manage machine learning and cryptography problems. PySyft [64] is a PyTorch-based library introduced by OpenMined. It enables privacy preservation in Deep Learning by using homomorphic encryption algorithms for individual data or model owners. The interested reader should also refer to the references [65,66].

## 5. Implementations of Some Homomorphic Encryption Toolkits

Academic and industrial companies are currently working hard to implement and deploy numerous homomorphic encryption libraries. In addition, many organizations are currently using or preparing to use fully homomorphic encryption libraries to build their data security applications. It appears that fully homomorphic encryption is very close to the real world for massive implementations and can mitigate the difficulties in the deployment phase. Table 2 lists the major homomorphic encryption tools. Most current libraries are designed for cryptographers and data analysts, and provide configurable capabilities that make it easy to explore the HE scheme and its parameters. From Table 2, we can see that there are simple criteria that can be applied when choosing which tool to use based on the computational efficiency of multiplicative homomorphic operations and the operating system. Existing libraries in Table 2 contain a collection of homomorphic operations that can be used to construct complicated functions. In this paper, we list the following primary libraries based on their usability and performance, which are known from previous research and the relevant existing research [28].

- **SEAL** is an open-source (MIT license) homomorphic encryption technology developed by Microsoft. The open-source library is in C++ and implements different homomorphic encryption algorithms, e.g., BGV and CKKS.
- **HElib** is IBM’s open-source cross-platform platform that facilitates several kinds of homomorphic encryption. The open-source library in C++ also implements different homomorphic encryption algorithms with various optimizations, e.g., BGV and CKKS.
- **Palisade** homomorphic encryption software library is a cross-platform open-source tool and library that provides various lattice cryptography implementations with construction blocks and homomorphic encryption algorithms. The open-source toolkit is based on C++ and supports several homomorphic encryption algorithms such as BFV, BGV, and CKKS.

Table 3 lists the average execution time (in microseconds) for the homomorphic encryption libraries of CKKS. All tests were run on an Intel i7-10710u CPU @ 1.61 Ghx and 16 GB from RAM running Ubuntu 20.04.3 LTS. The goal of this test was to compare the execution times of different cryptographic methods by measuring the duration for parameter creation, key generation, encryption, evaluation, and average ciphertext and decryption algorithms. In this context, we measured the execution time of the operations of the homomorphic encryption tools. First, we generated a random plaintext with a different ciphertext modulus and encrypted it into ciphertext. Then we applied the scoring function and finally decrypted the results. Each process was analyzed under the default parameters of each toolkit and in the same environment.

In this analysis, we evaluated the security level (128 bits) of each component and saw how the performance of the execution speed changed in seconds. We suspect that the differences in results between these tools are due to differences in programming language implementation and optimization in each tool. Furthermore, this comparison test took the default parameters of each homomorphic encryption algorithm in each tool and the same length of the plaintext.

**Figure 7:**
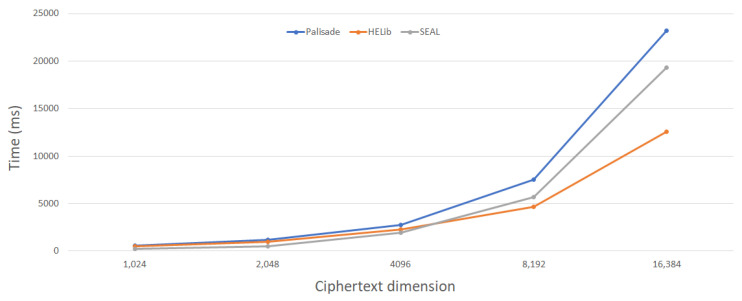
Comparison results of the runtime of the encryption algorithm based on the dimension of the ciphertext.

**Figure 8:**
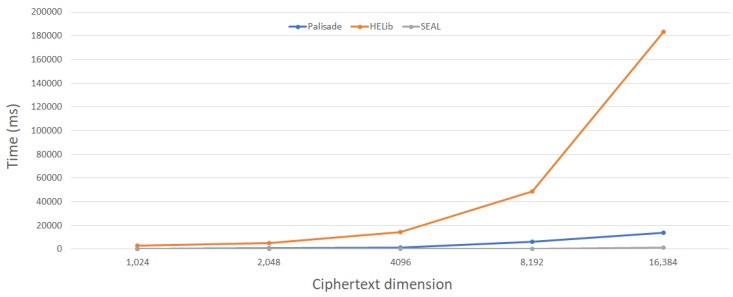
Comparison results of the runtime of the decryption algorithm based on the dimension of the ciphertext.

Figure 7 and Figure 8 show the results of the measured runtimes for encryption and decryption depend on the dimension of the ciphertext. As shown, the ratio of the performance differences decreases as the dimension of the ciphertext increases. The encryption process in the CKKS scheme performs best when the HELib library is used for ciphertext dimension n = 8192, but the SEAL library gives the best results for smaller dimensions n. The decryption process in the CKKS scheme performs best when the SEAL library is used. Using SEAL, the decryption procedure in the CKKS scheme is faster than using Palisade and HELib.

While the comparison in Table 3 appears to be simple, it provides information not only on how these tools perform but also on the performance speeds of the libraries. The BFV and CKKS schemes are supported in the SEAL and PALISADE libraries, while the BGV scheme is supported in the HElib and PALISADE toolkits. From the results of the implementation, it is clear that both encryption and decryption operations (with the public key and private key) have approximately the same performance in each homomorphic encryption algorithm.

Note that there are several limitations and challenges to fair comparisons; a specific comparison circuit should be used. For example, to create a ciphertext consisting of only zeros and ones in SEAL and HElib, a binary encoder must be used for all other tools. In reality, however, this approach can result in two similar ciphertexts that can be compared bit for bit. This procedure is both time consuming and insecure, according to Sathya et al. [67]. A machine with limited resources can decrypt the ciphertext by randomly comparing it to a known ciphertext. Because of these potential security threats, most homomorphic encryption tools and libraries do not readily provide a comparison API [67].

## 6. Discussion and Challenges

Although fully homomorphic encryption currently has a wide range of uses, several applications are currently unable to use fully homomorphic encryption due to the limitations of this technique. [68]. In general, homomorphic encryption applications are client-server scenarios where both the data and the method must be kept secret, and most current research does not address the security of the private keys. Here, it depends heavily on the context of the application itself.

One of the major obstacles in this context is the development of use cases for multimedia data, e.g., high-resolution images and videos [69,70,71], as fully homomorphic encryption is still quite slow due to insufficient computational power and the highly complex operations of homomorphic encryption algorithms. For example, an online face recognition application could only handle a few seconds delay. On the other hand, offline operations can be accommodated in homomorphic encryption applications, such as statistics on medical research results, even if this takes a considerable amount of time.

The homomorphic encryption algorithm is not without drawbacks; in addition to the known computational costs, we must consider two other factors. One example is the lack of multi-user functionality in most common homomorphic encryption algorithms. In theoretical studies, we assume that all data owners are encrypted with the same key, but this is rarely the case when there are numerous data owners in a practical scenario. In this context, multiple data owners may or may not trust each other. These data owners are not willing to train a model on jointly encrypted data. In the practical scenario, this situation can be solved by using a homomorphic encryption algorithm with multiple keys, which guarantees the encryption of the data with multiple independent keys and allows the trained model to be recovered from the different data owners, each having their own key.

One of the drawbacks is that homomorphic encryption requires high structural changes and specialized client-server applications to work properly. Industrial companies cannot use this technique for scientific analysis without first obtaining user consent. This can drive up overall costs and constantly divert the company’s attention to other solutions that are more efficient at encrypting analytics activities and ignore privacy concerns.

Most implementations of homomorphic encryption software, as well as various toolkits, have poor performance in terms of decrypted data quality when using current CPUs. This problem is actually an inherent problem resulting from the use of noise in lattice-based encryption algorithms. Unlike traditional encryption methods, FHE does not guarantee data integrity. Unlike traditional encryption methods, such as AES block-cypher or chaotic encryption, homomorphic encryption algorithms do not guarantee data integrity [72]. The problem of data corruption or integrity loss in homomorphic encryption tools can limit the effectiveness and accuracy of any Big Data analytics framework for privacy preservation.

Cryptoscientists are interested in discovering and predicting complex security and privacy situations in the real world based on cross-data analysis of various IoT Big Data sources [65]. For example, in federated learning, we need to use homomorphic encryption to protect the system and user privacy [45,59,73]. As we know from the current literature, federated learning systems are vulnerable to attacks from malicious clients [74,75]. Although the federated learning approach has been proposed to enable collaborative training, the privacy problem has increased with the risk of leaking collaborative data by combining different databases, especially against a variety of privacy attacks, such as the privacy threat from linkages between databases [1,76]. Given the high-impact privacy threat in federated learning systems, researchers may need to explore some defense strategies for the federated learning aggregator to identify malicious participants based on their model updates. Malicious attackers can potentially penetrate the computing infrastructure [77].

Due to the variety, volume, truth content, importance, and complexity of Big Data, data processing systems with ever-increasing computational capacities are needed. In addition, homomorphic encryption provides immutable, secure, and transparent data transfers that require more processing capacity. However, when sophisticated big data is encrypted with a homomorphic encryption algorithm, the system suffers from unexpected computational complexity, resulting in poor performance. Therefore, the structure of the homomorphic encryption algorithm should be further improved to meet the use cases and adaptive multi-key encryption.

Finally, it should be noted that we have only considered homomorphic encryption tools in our survey and discussion. Serious challenges in real industrial applications are often even more complicated and require a mix of approaches such as homomorphic encryption and multiparty computation techniques. Current tools have successfully minimized the complexity of complex homomorphic encryption algorithms. There is a wide range of libraries that enable secure and efficient implementation of current homomorphic encryption algorithms. Accordingly, we expect a more holistic approach to these challenges, and tool building will support a wider range of approaches that could be a significant asset to the secure computing industry. The scope of industrial use cases will grow in step with advances in the understanding and efficiency of homomorphic encryption algorithms. This essentially transformative solution will become more widespread for various Big Data applications, fundamentally changing the framework for how and where companies can leverage various private information assets.

## 7. Conclusions

In this paper, we presented an overview of privacy-preserving techniques in Big Data analytics with homomorphic encryption algorithms. We evaluated the security of homomorphic encryption and its lack of IND-CCA security. We also described an application scenario that may be of interest for analysis techniques between data owners and data analysts. Then, we listed and compared the features and performance of several new homomorphic encryption toolkits. Finally, we examined the obstacles and gaps in deploying practical, secure applications in the field. The results of this work are relevant to both academics and industrialists working in the area of encryption-based Big Data analytics. Based on the research results, we offer some theoretical and practical implications for integrating advanced encryption technology into Big Data applications. A homomorphic encryption algorithm is a viable approach for industrial applications that enable secure and efficient collaboration between data owners and untrusted data analyzers.

Due to the fact that this is a fruitful area of research, we anticipate that further discoveries will enable broader use of private data in production environments, especially to support Big Data analytics processes.

## Figures and Tables

**Table 1:** Well-known partially homomorphic encryption algorithms’ properties.

Scheme	Homomorphic	Operation
	**Add**	**Mult**
RSA (Rivest et al., 1978)		🗸
El-Gamal (ElGamal 1985)		🗸
Paillier (Paillier 1999)	🗸	
DJ (Damgård and Jurik 2001)	🗸	
Galbraith (Galbraith 2002)	🗸	
KTX (Kawachi et al., 2007)	🗸	

**Table 2:** Homomorphic Encryption Toolkits.

Library	Author & Schemes	Language	Initial Release	Last Major Update	Software License
HElib	Halevi, Shoup (IBM), BGV, CKKS	C++	May 2013	August 2019	Apache v2.0
HEAAN	Cheon, Kim, Kim, Song, CKKS	C++	May 2016	July 2021	CC BY-NC 3.0
PALISADE	NJIT, BFV, BGV, and CKKS	C++	July 2017	August 2021	BSD 2-clause
TFHE	Chillotti et al., TFHE	C++	April 2017	February 2020	Apache v2.0
Microsoft SEAL	Microsoft, BFV, CKKS	C++	December 2018	November 2020	MIT
NuFHE	NuCypher, GPU based TFHE	Python	October 2018	July 2019	GPL-3.0
Lattigo	EPFL-LDS, BFV, CKKS	Go	December 2020	July 2021	Apache v2.0

**Table 3:** Average of the execution time (in microseconds) for CKKS homomorphic encryption algorithms.

*n*	Palisade	HELib	SEAL
1024	585	482	257
1024	415	3159	10
2048	1173	997	479
2048	809	5104	19
4096	2753	2288	1926
4096	1432	14,279	72
8192	7538	4664	5688
8192	6038	48,960	290
16,384	23,183	12,581	19,344
16,384	13,776	183,254	1166

## Data Availability

Data sharing not applicable.
